# T Cell Response in Patients with Implanted Biological and Mechanical Prosthetic Heart Valves

**DOI:** 10.1155/2016/1937564

**Published:** 2016-02-17

**Authors:** L. Barbarash, I. Kudryavtsev, N. Rutkovskaya, A. Golovkin

**Affiliations:** ^1^Federal State Budgetary Institution Research Institute for Complex Issues of Cardiovascular Diseases, Kemerovo 650002, Russia; ^2^Federal State Budgetary Institution Research Institute for Experimental Medicine, Saint Petersburg 197376, Russia; ^3^Far Eastern Federal University, Vladivostok 690091, Russia; ^4^Federal Almazov Medical Research Centre, Saint Petersburg 197341, Russia

## Abstract

The study was aimed at assessing T cell subsets of peripheral blood from recipients of long-term functioning (more than 60 months) biological and mechanical heart valve prostheses. The absolute and relative number of CD4 and CD8 T cell subsets was analyzed: naïve (N, CD45RA^+^CD62L^+^), central memory (CM, CD45RA^−^CD62L^+^), effector memory (EM, CD45RA^−^CD62L^−^), and terminally differentiated CD45RA-positive effector memory (TEMRA, CD45RA^+^CD62L^−^) in 25 persons with biological and 7 with mechanical prosthesis compared with 48 apparently healthy volunteers. The relative and absolute number of central memory and naïve CD3^+^CD8^+^ in patients with biological prosthesis was decreased (*p* < 0.001). Meanwhile the number of CD45RA^+^CD62L^−^CD3^+^CD8^+^ and CD3^+^CD4^+^ was increased (*p* < 0.001). Patients with mechanical prosthesis had increased absolute and relative number of CD45RA^+^CD62L^−^CD3^+^CD8^+^ cells (*p* = 0.006). Also the relative number of CD3^+^CD4^+^ cells was reduced (*p* = 0.04). We assume that altered composition of T cell subsets points at development of xenograft rejection reaction against both mechanical and biological heart valve prostheses.

## 1. Introduction

At present, selecting a type of prosthetic heart valve for surgical correction of acquired cardiac failure represents a topical issue for modern medicine. Biological or mechanical prosthetic heart valve are available options. In particular, xenogeneic tissue treated by fixatives and preserving solutions is applied in the former, whereas in the latter various synthetic materials were applied (plastic, polymers, etc.).

Perhaps one of the criteria for selecting type of prosthesis might be a response of immune system to implanted xenogeneic material. Indeed, a foreign body is literally placed into the blood flow, which constantly contacts with blood cells and surrounding tissues and could eventually cause inflammatory and, perhaps, autoimmune reactions. Character and intensity of such reactions are subject to thorough investigation in order to, on one hand, select proper prosthetic heart valve and, on the other hand, develop approaches for improving prostheses and preventing complications and their dysfunctions. Previously, it was found that biological compared to mechanical prostheses may cause inflammatory complications [1]. Despite this, other data from long-term studies revealed no significant differences between biological and mechanical prostheses in terms of subsequent complications [2].

Previously, types of immune response developed to implanted prosthesis were discussed as well. For this, a range of cells infiltrating prosthesis was examined. It was demonstrated that T cells infiltrate pannus while investigating removed biological and mechanical prostheses. Upon that, quantity of the cells found inside infiltrate differed and varied depending on intensity of local inflammatory response. Cellular components were presented by CD4^+^ and CD8^+^ T lymphocytes [3].

Examination of the removed mechanical prostheses demonstrated that they contained more CD15^+^, CD68^+^, CD3^+^, Factor VIII^+^ cells upon verified infectious etiology of prosthetic failure compared to infection-free samples [4]. In case of lacking infection accompanied by a marked fibrosis, inflammatory infiltrates mainly containing lymphocytes and macrophages were developed.

Similar comparative examination of the removed biological prosthetic heart valves demonstrated that profound infiltration of prosthetic tissues with macrophages (CD68) and T cells (CD3) occurs in case of any prosthetic dysfunction [5].

By analyzing 17 removed Medtronic Freestyle bioprostheses with average implant duration of 71.1 ± 35.2 months it was found that signs of chronic inflammatory reaction affecting the xenograft arterial wall were observed in 15 cases. Infiltrates consisted of macrophages and lymphocytes including B and T cells. During the study, it was concluded that T cells responded to implanted foreign porcine tissues with development of significant damage of host aortic wall. It is assumed that inadequately fixed tissues of porcine aorta resulting in subsequently retained antigenicity might be one of potential causes of developing inflammation [6]. A hypothesis is confirmed regarding causes underlying development of immune reactions against implanted biological tissue related to residual antigens of animal origin retained despite special treatments and decellularization [7, 8].

It becomes evident that both mechanical and biological prosthetic heart valves are associated with tissue reactions developing against implanted foreign materials. Upon that, macrophages, neutrophils, and T cells are the major players of cellular immunity in this process. However, dynamic changes of their quantities in systemic blood flow, participation in pathogenesis of responses against foreign materials, and potential diagnostic importance were poorly investigated.

In connection with this, our study was* aimed at* assessing T cell subsets of peripheral blood from recipients of long-term functioning biological and mechanical heart valve prostheses.

## 2. Materials and Methods

Current study was performed with 32 recipients of prosthetic heart valves. All patients underwent replacement of mitral valve including 25 persons with biological and 7 with mechanical prosthesis. There were used biological prostheses prepared from swine aortic valve. Characteristics of patients, etiology of heart defect, comorbidities, and duration of prosthesis functioning are presented in Table 1. Surgical treatment and follow-up were done at the Research Institute For Complex Issues Of Cardiovascular Diseases (Kemerovo, Russia). All patients underwent follow-up examination every six months after surgical correction.

In comparison group 48 apparently healthy volunteers were included. No differences in age and gender composition were found between patients and healthy volunteers.

All persons enrolled to the study were properly notified and signed an informed consent.

### 2.1. Collection of Blood Samples and Preparation

Samples of peripheral blood from ulnar vein were collected from patients and healthy volunteers into test tubes containing К_3_EDTA. To characterize T cell phenotypes the following combinations of monoclonal antibodies (Beckman Coulter, USA) were used: (1) anti-human CD45RA-FITC (cat. A07782, clone J.33), anti-human CD62L-PE (cat. IM2214U, clone DREG56), anti-human CD8-PC5.5 (cat. A99049, clone T8), and anti-human CD3-APC (cat. IM2467, clone UCHT1); (2) anti-human CD45RA-FITC (cat. A07782, clone J.33), anti-human CD62L-PE (cat. IM2214U, clone DREG56), anti-human CD8-PC5.5 (cat. A99049, clone T8), anti-human CD3-APC (cat. IM2467, clone UCHT1), and anti-human CD4-PC5.5 (cat. B16491, clone 13B8.2).

Briefly, 100 *μ*L of peripheral blood cells was stained with each combination of antibodies according to the manufacturer's instructions for 15 minutes in the dark, at room temperature, followed by lysing red blood cells with VersaLyse Lysing Solution (cat. A09777, Beckman Coulter, USA). The absolute number of leukocytes, lymphocytes, granulocytes, and monocytes was counted by using hematology analyzer MEK-6400 (Nihon Kohden, Japan).

### 2.2. Flow Cytometry Analysis

Stained samples were analyzed by running a four-color flow cytometry with FACSCalibur (Becton-Dickinson, USA) and Navios (Beckman Coulter, USA). Mathematical processing of the flow cytometry data was performed by using Kaluza*™* v.1.2 (Beckman Coulter, USA) software.

A threshold was set to a forward-scatter (FSC) parameter to exclude cell debris. The SSC and FSC settings were done with linear amplification and the logarithmic amplification scale was used for the fluorescence channels and dot plot analysis. Isotype-matched control abs. and “fluorescence-minus-one” gating techniques were used to evaluate thresholds for positivity of individual antibodies. The following CD4 and CD8 T cell subsets were analyzed: naïve (N, CD45RA^+^CD62L^+^), central memory (CM, CD45RA^−^CD62L^+^), effector memory (EM, CD45RA^−^CD62L^−^), and terminally differentiated CD45RA-positive effector memory (TEMRA, CD45RA^+^CD62L^−^) according to CD45RA and CD62L expression.

**Figure 1:**
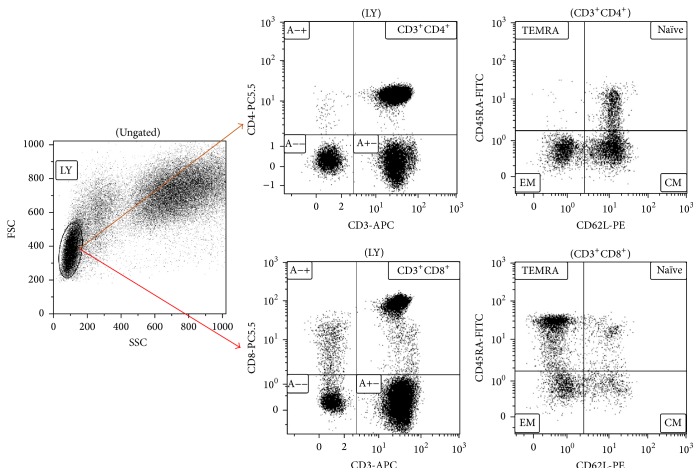
Representative example of multicolor flow cytometry analysis after gating on CD4 or CD8 T cells from lymphocyte population. Comments: naïve (N), central memory (CM), effector memory (EM), and terminally differentiated CD45RA-positive effectors (TEMRA).

Lymphocytic cells were gated according to forward-scatter (FSC) and side-scatter (SSC) properties. Representative bivariate dot plots of the isolated lymphocyte populations from human peripheral blood samples are presented. CD3^+^CD4^+^ T helper and CD3^+^CD8^+^ cytotoxic T cells are shown on dot plots, respectively. Immunophenotyping was done by analyzing flow cytometry data after costaining with CD3 and CD4 or CD3 and CD8 cell surface markers on FSC/SSC gated lymphocytes, respectively (Figure 1).

### 2.3. Statistical Analysis

Statistical analysis of the data was done by using Statistica 7.0 and GraphPad Prism software. The data are presented as median (Ме) values and interquartile range (25; 75%). Significance of differences between data was evaluated by applying nonparametric Mann-Whitney *U* test and *W*-Wilcoxon test. A correlation analysis was done by using Spearman *r*-test. Significance level was set at *p* < 0.05.

Multivariate comparison was done by using discriminate analysis. A stepwise analysis enumerating steps, *p* value significance level, and *F*-test were performed. A discrimination level was evaluated by assessing Wilks' lambda. Significance of an identifying criterion was determined after drawing scatterplots of canonical values and calculating classification value and Mahalanobis squared distance.

## 3. Results

Parameters of hemogram (WBC total, count of neutrophils, lymphocytes, and monocytes) were shown to lack differences between patients with biological and mechanical heart valve prostheses as well as healthy volunteers (Table 2).

While examining T cell arm of immunity it was found that recipients with biological and mechanical heart valve prostheses did not differ in terms of both relative and absolute counts of all examined T cell subsets. At the same time, these parameters were shown to differ when compared with healthy volunteers (Table 3).

Recipients of both mechanical and biological heart valve prostheses were found to have reduced counts of CD3^+^ cells compared to healthy volunteers. Patients with biological prostheses had significantly lower relative and absolute amounts of CM Tcyt and naïve Tcyt compared to healthy volunteers; conversely, amount of TEMRA Tcyt and TEMRA Th cells was elevated.

Patients with mechanical heart valve prostheses were documented to have increased relative and absolute amount of TEMRA Tcyt compared to healthy volunteers. In addition, relative amount of Th (CD3^+^CD4^+^) cells was also found to decline compared to healthy volunteers.

In contrast, no significant differences were found in examined parameters of T cell subsets from patients with biological heart valve prostheses versus healthy volunteers. Moreover, no correlation between hemogram parameters and duration of prosthesis functioning was revealed as well.

**Figure 2:**
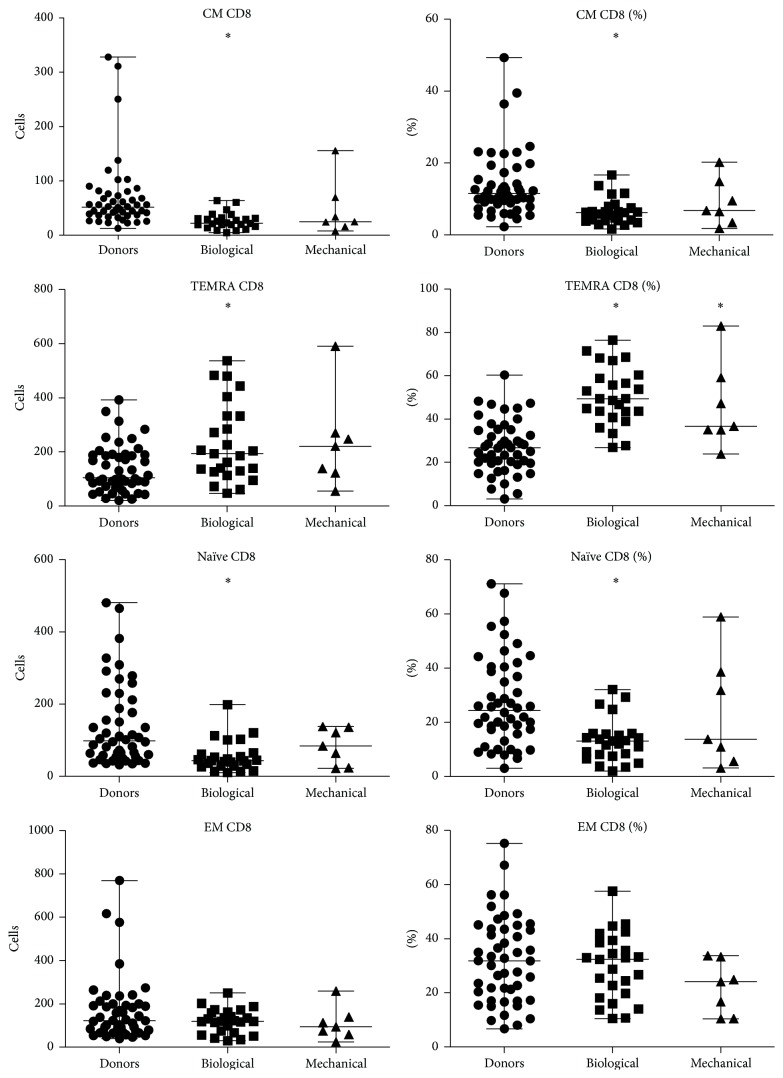
Representative pattern of CD8 T cell subsets among patients with mechanical versus biological heart valve prostheses. ^*∗*^Significant difference with healthy donors *p* < 0.01.

**Figure 3:**
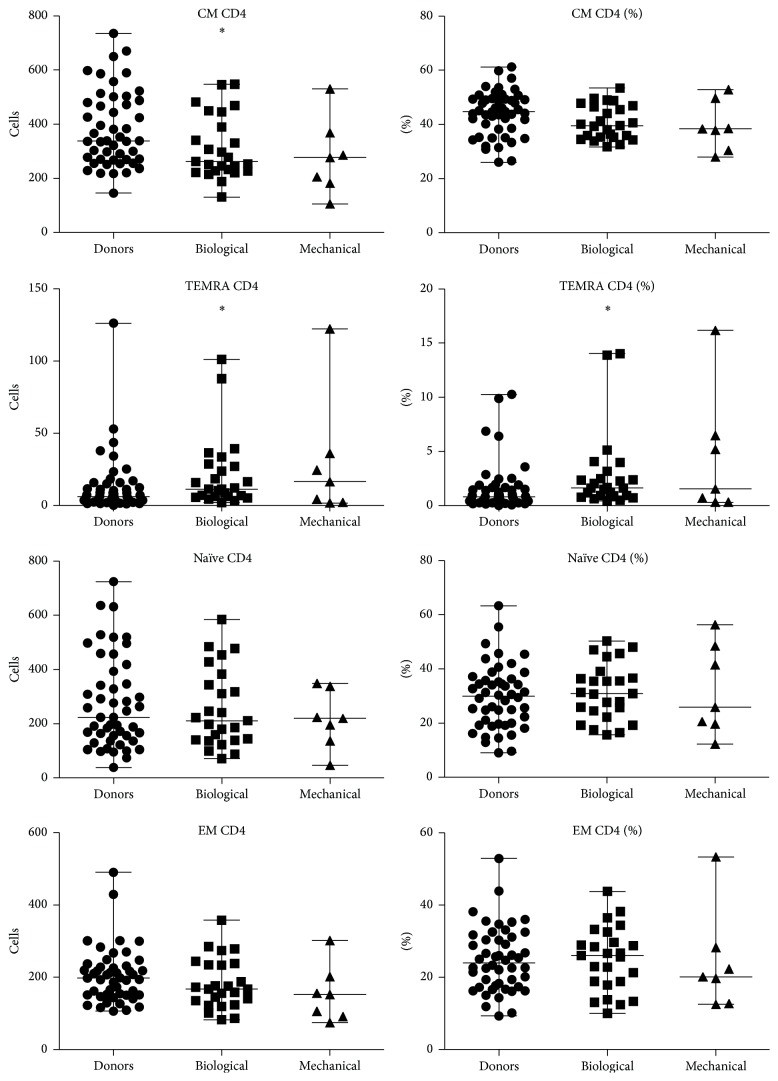
Representative pattern of CD4 T cell subsets among patients with mechanical versus biological heart valve prostheses. ^*∗*^Significant difference with healthy donors *p* < 0.01.

Representative distribution of T cell subsets among patients with mechanical versus biological heart valve prostheses is shown in Figures 2 and 3.

**Figure 4:**
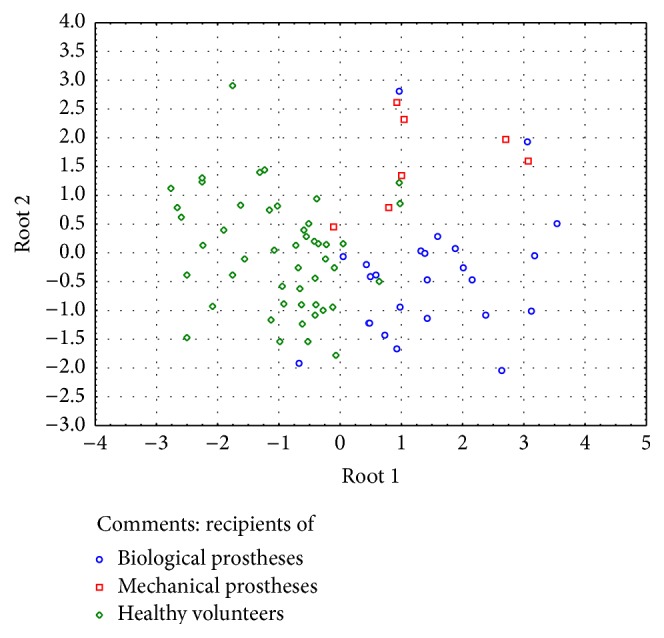
Graphic distribution of patient groups according to discriminate analysis.

A discriminant analysis done using a forward stepwise model consisting of 7 steps demonstrated the highest significance level while verifying counts of TEMRA Tcyt relative, CD3^+^ relative, naïve Th relative, EM Tcyt relative, CD3^+^CD8^+^ relative, naïve Tcyt abs, and TEMRA Th relative (Table 4). Partition of the examined groups based on the results of discriminant analysis is depicted in Figure 4.

Only in group of biological prosthesis recipients it was possible to analyze the effect of comorbidities to the changes in T cell subpopulations. There were no significant changes in patients with or without chronic ischemic heart disease, diseases of the urinary system, pulmonary, and thyroid diseases. All significant differences between biological prosthesis recipients with comorbidities (diabetes, hypertonic disease, acute cerebrovascular accident, and GI-tract diseases) are presented in the Tables 5, 6, 7, and 8.

## 4. Discussion

Among all surface markers used in our study, various CD45 isoforms had the longest history of practical application. As early as in 1988 it was demonstrated that CD45R (now known as CD45RA) protein may be considered as a marker for naïve or unprimed T cells, whereas UCHL1 antibody recognizing CD45R0 binds to memory T cells [9]. Currently, it is known that naïve T cells express CD45 molecules containing all domains within its sequence; however, starting from antigen-specific differentiation maturing T cells begin to express their isoforms resulting from mRNA splicing within exon A followed by exons B and C. Gene product containing all these domains is known as CD45RA (molecular weight 220 kDa), whereas the product derived after final RNA modification and lacking all such domains is denoted as CD45R0 (180 kDa). Currently, functional significance of various CD45 isoforms remains poorly investigated, which is not true for the rest of surface markers used for phenotyping main stages of maturing T cells [10].

Picker et al. [11] first described CD62L (L-selectin) as a molecule determining direction of migrating naïve T cells trafficking into peripheral lymphoid tissues. Moreover, they also demonstrated that naïve T cells (CD45RA^high^/R0^low^) mainly expressed CD62L, whereas more mature CD45RA^low^/R0^high^ might be separated as a cell population being both CD62L^+^ and CD62L^−^. The latter subset bears adhesion molecules responsible for cell migration into body peripheral tissues. Currently, it is considered that both CD62L and CCR7 markers determine migration of CD3^+^CD4^+^ and CD3^+^CD8^+^ T cells from peripheral blood. By staining for CCR7 and CD62L molecules on the cell surface it allows to denote naïve and central memory T cells within the pool of circulatory T lymphocytes [12]. Lack of such surface markers on effector T cells—effector memory and terminally differentiated effector T cells (TEMRA)—is accounted for by the fact that these cell subsets function within nonlymphoid tissues.

Central memory T cells are characterized by surface expression of CD45R0 instead of CD45RA as well as CD62L, CD27, CD28, and so forth. The main difference of this T cell subset from naïve T cells is that they have already passed through antigen-specific differentiation that occurred within the secondary lymphoid tissues. Presence of CD62L on the surface of these cells allows to distinguish them from effector memory T cells with phenotype CD45RA^−^CD62L^−^. This feature of Tcm allows them to circulate around the body for a long period of time and determines their preferential location inside the secondary lymphoid tissues. Upon antigenic stimulation, antigen presenting cells possessing cognate surface ligands allow to rapidly activate T cells with high expression level of CD27 and CD28 followed by successful formation of antigen-specific T cell clones [13]. Moreover, central memory T cells better secrete IL-2, whereas effector memory T cells are more effective in synthesizing effector cytokines [14].

Previously, a role for T cell subsets in rejecting grafted organs was investigated. In particular, 47 out of 185 patients with morphologically verified acute rejection reaction of transplanted kidney also compared with healthy volunteers were found to possess more differentiated T cells at terminal stage of chronic renal failure. In addition, patients with acute rejection reaction were noted to have signs of dysregulated T cell profile and bear elevated amount of total T cells including naïve T cells but lowered count of terminally differentiated memory T cells. However, functional assays demonstrated that the latter subset had upregulated proinflammatory and cytotoxic capacity [15].

In another study, 131 patients with normally functioning transplanted kidney were examined. Among them, increased amount of terminally differentiated memory T cells in 45 patients was associated with restricted TCR V*β* repertoire (CD45RA^+^CCR7^−^CD27^−^CD28^−^CD8^+^). In 47 patients graft dysfunction (median age = 15 years) was documented. A 2-fold increased risk of developing graft dysfunction was observed in patients with elevated amount of TEMRA CD8^+^ T cells [16].

It was found that patients at reactive stage of kidney graft rejection had elevated relative count of memory CD4 (TEM) and terminally differentiated CD8 (TEMRA) T cells compared to patients at quiescence stage and healthy volunteers. In case of acute rejection, a significant decrease in count of CD8 TEMRA was documented [17].

High diagnostic significance of increased level of D8 TEMRA and reciprocally decreased naïve T cells was observed in patients after bone marrow transplantation and development of chronic graft versus host disease [18].

Investigation of T cell subset repertoire in peripheral blood from patients with implanted biological or mechanical heart valve prostheses demonstrated that T cells strongly responded to foreign material. At that, despite the fact that no significant differences between all examined parameters of T cell immunity were found in recipients of different types of prostheses, some of them, however, significantly differed when compared to healthy volunteers.

We assume that altered composition of T cell subsets, namely, decreased counts of CM Tcyt, naïve Tcyt paralleled with elevated amount of Emra Tcyt, and Emra Th, points at development of xenograft rejection reaction against both mechanical and biological heart valve prostheses. Importantly, a more pronounced rejection reaction was against biological prostheses. It seems that despite special treatment of biological material in graft tissues, quite a large amount of xenogeneic tissue antigens still remains (swine valve apparatus). T cells and monocytes become stimulated by foreign antigens that also provoke their migration into donor tissues and* in situ* activation resulting in release of huge amounts of proinflammatory cytokines. In particular, a whole set of such biologically active molecules may display proosteogenic activity not only supporting local inflammatory reaction, but also leading to collagen degradation and deposition of hydroxyapatites. Altogether, local inflammatory and degenerative changes within prosthetic tissues may result in prosthesis dysfunction [19].

Despite the fact that no correlation between changes in T cell subset repertoire and signs of prosthesis dysfunctioning as well as duration of prosthesis functioning was found, however, it may be assumed that immune cells are involved in developing local changes within prosthetic tissues. At the same time, results of discriminant analysis suggest that T cell subsets from recipients both of biological and mechanical heart valve prostheses display distinguishing signs of response against graft tissues. Moreover, in the future it might be possible to determine diagnostically relevant parameters of T cell subset properties to be used for early diagnostics of host-versus-graft reaction.

Unfortunately there are only few articles describing peripheral T cell subset changes in heart disease as well as in other comorbidities. Nevertheless we tried to find out their impact on T cell subset compositions.

Studying patients with acute myocardial infarction undergoing primary percutaneous coronary intervention has found that cytomegalovirus-seropositive patients demonstrated a greater fall in the concentration of terminally differentiated CD8 effector memory T cells in peripheral blood during the first 30 minutes of reperfusion compared with cytomegalovirus-seronegative patients. Moreover a significant proportion of TEMRA cells remained depleted for ≥3 months in cytomegalovirus-seropositive patients. Hereby myocardial ischemia and reperfusion in cytomegalovirus-seropositive patients lead to acute loss of antigen-specific, terminally differentiated CD8 T cells [20].

Type 1 diabetes is an autoimmune process and has other pathogeneses pathways compared to type 2. Nevertheless while studying 55 patients with type 1 diabetes it was found that percentages and absolute numbers of CM and N cells were reduced, whereas those of TEMRA cells were markedly increased. The indices of intermediate- and long-term glycaemic control were associated negatively with the number of CM and N cells while positively with the number of TEMRA cells. Authors conclude that considerable accumulation of TEMRA T cells suggests lifelong stimulation by protracted antigen exposure (viruses, other agents, or residual self-antigens) or a homeostatic defect in the regulation/contraction of immune responses [21].

Studying T-lymphocyte subsets in patients with severe acute respiratory syndrome (SARS) investigators found that cell count of naïve CD4^+^ (CD4^+^CD45RA^+^CD62L^+^) remarkably decreased during the 1st week after the infection. During the 8th–12th weeks, the cell counts of naïve CD4^+^ subset were still less than those of normal controls, while comparing with those of the 1st week. Authors conclude that it will take more than 8–12 weeks for CD4^+^ cell and naïve CD4^+^ subset to reach to normal levels after SARS [22].

In our research all founded significant differences in patients with and without comorbidities (diabetes, hypertonic disease, acute cerebrovascular accidents, and GI-tract diseases) had the common trend. We hypothesize that any chronic diseases could lead to changes in T cell populations because of the participation of immune system. Meanwhile decreased relative and absolute number of central memory and naïve CD3^+^CD8^+^ and increased number of CD45RA^+^CD62L^−^CD3^+^CD8^+^ and CD3^+^CD4^+^ in patients with biological prosthesis were in the common trend regardless of the presence of any comorbidities. That is why we assume that these changes are related to the development of xenograft rejection reaction.

## 5. Limitations

Current study contains the following limitations: the number of examined patients with implanted mechanical prostheses was low (7 persons); duration of prosthesis functioning was significantly shorter in this group compared to patients with biological heart valve prostheses. Taking together these limitations may lead to the loss of the significant differences of the patients with mechanical prosthesis comparing with recipients of biological prosthesis and healthy volunteers.

## Figures and Tables

**Table 1:** Clinical characteristics of patients.

Characteristics	Biological prosthesis, *n* = 25	Mechanical prosthesis, *n* = 7
Age of patient at the time of primary surgery	53 (49; 57)	54 (28; 62)
Male/female (*n*/*n*)	7/18	1/6
%	28/72	14.3/85.7
Duration of prosthesis functioning, month	100 (83; 116)	70 (64; 87)^*∗*^
Etiology of heart defect		
Rheumatic heart disease	19 (76%)	6 (85.7%)
Infective endocarditis	3 (12%)	0
Connective tissue dysplasia	3 (12%)	1 (14.3%)
Functioning of prosthesis		
Sustained	18 (72%)	7 (100%)
Signs of malfunction	7 (28%)	0
Heart failure (NYHA)		
I	9 (36%)	2 (28.6%)
IIА	16 (64%)	5 (71.4%)
Functional class of heart failure		
1	1 (4%)	1 (14.3%)
2	19 (76%)	5 (71.4%)
3	4 (16%)	1 (14.3%)
4	1 (4%)	0
Cardiac dysrhythmia		
Sinus rhythm	7 (28%)	4 (57.1%)^*∗∗*^
Paroxysmal atrial fibrillation	5 (20%)	3 (42.9%)^*∗∗*^
Permanent atrial fibrillation	12 (48%)	
Ectopic heartbeat	1 (4%)	
Hypertonic disease	7 (28%)	4 (57.1%)
Type 2 diabetes mellitus	3 (12%)	0
Chronic ischemic heart disease	2 (8%)	1 (14.3%)
Acute cerebrovascular accident	3 (12%)	1 (14.3%)
GI-tract diseases	13 (52%)	4 (57.1%)
Thyroid diseases	2 (8%)	1 (14.3%)
Diseases of the urinary system	5 (20%)	2 (28.6%)
Pulmonary diseases	3 (12%)	1 (14.1%)

Comments: ^*∗*^ *p* = 0.011, ^*∗∗*^ *p* = 0.03.

**Table 2:** Peripheral blood hemogram from patients with biological and mechanical heart valve prostheses and healthy volunteers.

Cell type	Healthy volunteers, *n* = 48	Biological prosthesis, *n* = 25	Mechanical prosthesis, *n* = 7
WBC	6.0 (5.1; 7.5)	6.0 (5.4; 7.0)	5.3 (3.9; 7.1)
Neutrophils	3.3 (2.8; 4.6)	3.5 (2.9; 4.7)	2.9 (2.0; 4.3)
Lymphocytes	1.9 (1.5; 2.3)	1.8 (1.6; 1.9)	1.6 (1.3; 1.7)
Monocytes	0.4 (0.3; 0.5)	0.5 (0.4; 0.6)	0.5 (0.4; 0.6)

**Table 3:** Profile of T cell subsets of peripheral blood from recipients with biological and mechanical heart valve prostheses and healthy volunteers.

T cells populations	Healthy donors, *n* = 48	Biological prosthesis, *n* = 25	Mechanical prosthesis, *n* = 7
CD3^+^%	78.27 (73.07; 82.52)	69.87 (65.68; 76.34)^*∗∗*^	69.96 (64.03; 71.72)^*∗*^
CD3^+^ abs.	1419.31 (1126.25; 1735.91)	1275.84 (1004.40; 1482.00)	1088.51 (977.34; 1185.92)
CD3^+^CD8^+^%	25.80 (20.64; 33.20)	24.28 (18.45; 27.32)	27.48 (24.58; 30.79)
CD3^+^CD8^+^ abs.	462.37 (349.70; 647.87)	412.00 (297.12; 597.77)	417.86 (351.00; 712.64)
CM Tcyt%.	11.50 (8.70; 16.38)	6.19 (3.93; 7.50)^*∗∗*^	6.75 (3.39; 14.85)
CM Tcyt abs.	51.69 (36.70; 74.30)	22.28 (18.98; 30.64)^*∗∗*^	24.63 (15.77; 69.37)
EM Tcyt%	31.82 (18.80; 43.55)	32.36 (19.75; 38.57)	24.14 (10.52; 33.38)
EM Tcyt abs.	122.56 (74.29; 196.04)	119.62 (74.36; 158.91)	94.26 (58.96; 139.48)
TEMRA Tcyt%	26.67 (19.58; 34.93)	49.41 (43.46; 58.83)^*∗∗*^	36.72 (35.04; 59.20)^*∗∗∗∗*^
TEMRA Tcyt abs.	104.32 (72.94; 188.42)	193.40 (129.60; 332.12)^*∗∗*^	220.78 (122.99; 270.64)
N Tcyt%	24.46 (16.51; 39.58)	13.08 (8.10; 15.63)^*∗∗*^	13.75 (5.59; 38.64)
N Tcyt abs.	98.47 (56.29; 199.74)	43.46 (28.49; 61.35)^*∗∗*^	83.67 (23.35; 135.62)
CD3^+^CD4^+^%	48.06 (41.04; 52.58)	40.74 (36.60; 48.37)	42.73 (41.01; 44.93)^*∗*^
CD3^+^CD4^+^ abs.	835.92 (624.17; 1096.10)	717.82 (580.50; 967.40)	697.17 (535.34; 755.99)
CM Th%	44.67 (38.42; 49.32)	39.60 (35.30; 46.42)	38.36 (30.46; 49.67)
CM Th abs.	338.29 (269.64; 484.11)	262.64 (227.73; 389.99)^*∗∗∗*^	276.91 (182.58; 368.45)
EM Th%	24.02 (17.54; 30.71)	26.01 (18.83; 29.66)	20.13 (12.71; 28.25)
EM Th abs.	197.64 (150.63; 229.90)	167.11 (135.15; 234.18)	152.18 (91.37; 201.64)
TEMRA Th%	0.82 (0.37; 1.67)	1.62 (0.94; 2.49)^*∗∗*^	1.54 (0.32; 6.47)
TEMRA Th abs.	6.17 (3.11; 13.58)	11.24 (6.24; 26.89)^*∗∗*^	16.45 (2.22; 36.04)
N Th%	29.88 (19.81; 36.00)	30.91 (24.50; 36.61)	25.87 (19.65; 48.46)
N Th abs.	222.99 (145.88; 369.81)	211.18 (140.38; 343.04)	219.42 (136.99; 337.60)
CD4/CD8	2.01 (1.34; 2.44)	1.93 (1.39; 2.17)	1.49 (1.38; 1.81)

Comments: denoted T cell subsets: N: naïve, CM: central memory, EM: effector memory, TEMRA: terminally differentiated CD45RA-positive effector cells, Tcyt: T cytotoxic (CD3^+^CD8^+^), and Th: T helper (CD3^+^CD4^+^). ^*∗*^Difference between values compared to healthy volunteers, *p* = 0.04; ^*∗∗*^difference of parameter compared to healthy volunteers, *p* < 0.001; ^*∗∗∗*^difference between values compared to healthy volunteers, *p* = 0.002; ^*∗∗∗∗*^difference between values compared to healthy volunteers, *p* = 0.006.

**Table 4:** Results of discriminate analysis assessing parameters of T cell subsets in patients with mechanical and biological heart valve prostheses as well as healthy volunteers.

Parameter	Wilks' lambda	Partial lambda	*F*-remove (2,71)	*p* value
TEMRA Tcyt relative	0.429027	0.743609	12.24012	0.000027
CD3^+^ relative	0.424789	0.751029	11.76849	0.000039
Naive Th relative	0.374814	0.851165	6.20755	0.003277
EM Tcyt relative	0.373348	0.854507	6.04443	0.003766
CD3^+^CD8^+^ relative	0.380216	0.839072	6.80864	0.001972
Naive Tcyt abs.	0.373151	0.854960	6.02242	0.003838
TEMRA Th relative	0.346166	0.921605	3.01974	0.055125

Comments: Step 7, *n* of variants in the model 7; Wilks' lambda = 0.3190287; *F*(14.142) = 7.814625, *p* < 0.00001.

**Table 5:** Impact of diabetes to T cells populations in biological prosthesis recipients.

T cells populations	Healthy donors, *n* = 48	Diabetes, *n* = 3	No diabetes, *n* = 22	*p* value
CD3^+^%	78.27 (73.07; 82.52)	84.21 (65.83; 86.52)	69.26 (62.71; 74.10)	1–3 *p* < 0.001
CD3^+^ abs.	1419.31 (1126.25; 1735.91)	1515.78 (789.96; 1643.88)	1253.32 (1004.40; 1441.86)	n/s
CD3^+^CD8^+^%	25.80 (20.64; 33.20)	24.95 (24.76; 38.91)	23.45 (18.07; 27.32)	n/s
CD3^+^CD8^+^ abs.	462.37 (349.70; 647.87)	474.05 (297.12; 700.38)	408.55 (280.98; 597.77)	n/s
CM Tcyt%.	11.50 (8.70; 16.38)	6.41 (2.71; 7.50)	5.99 (3.93; 7.94)	1–3 *p* < 0.001 1-2 *p* = 0.02
CM Tcyt abs.	51.69 (36.70; 74.30)	22.28 (18.98; 30.38)	23.45 (16.15; 31.72)	1–3 *p* < 0.001 1-2 *p* = 0.006
EM Tcyt%	31.82 (18.80; 43.55)	42.60 (26.71; 45.43)	30.56 (18.08; 35.61)	n/s
EM Tcyt abs.	122.56 (74.29; 196.04)	187.07 (134.98; 201.94)	118.07 (66.12; 140.10)	2-3 *p* = 0.04
TEMRA Tcyt%	26.67 (19.58; 34.93)	43.62 (43.46; 68.62)	49.45 (40.86; 58.83)	1–3 *p* < 0.001 1-2 *p* = 0.009
TEMRA Tcyt abs.	104.32 (72.94; 188.42)	206.02 (129.60; 480.60)	189.40 (126.49; 332.12)	1–3 *p* = 0.007
N Tcyt%	24.46 (16.51; 39.58)	3.45 (1.96; 7.53)	13.49 (9.09; 15.83)	1–3 *p* < 0.001 1-2 *p* < 0.001 2-3 *p* = 0.006
N Tcyt abs.	98.47 (56.29; 199.74)	13.72 (10.25; 35.69)	45.06 (33.37; 64.76)	1–3 *p* < 0.001 1-2 *p* < 0.001 2-3 *p* = 0.02
CD3^+^CD4^+^%	48.06 (41.04; 52.58)	45.40 (37.69; 60.29)	40.72 (36.19; 48.37)	1–3 *p* = 0.04
CD3^+^CD4^+^ abs.	835.92 (624.17; 1096.10)	817.20 (452.28; 1145.51)	705.31 (580.50; 967.40)	n/s
CM Th%	44.67 (38.42; 49.32)	47.82 (36.38; 48.95)	39.46 (35.23; 45.49)	1–3 *p* = 0.04
CM Th abs.	338.29 (269.64; 484.11)	297.29 (221.39; 547.78)	257.76 (227.73; 389.99)	1–3 *p* = 0.01
EM Th%	24.02 (17.54; 30.71)	34.39 (21.28; 43.80)	25.82 (17.82; 28.86)	n/s
EM Th abs.	197.64 (150.63; 229.90)	243.76 (155.53; 357.93)	167.01 (123.98; 233.30)	n/s
TEMRA Th%	0.82 (0.37; 1.67)	2.26 (0.94; 3.17)	1.62 (0.90; 2.49)	1–3 *p* = 0.006
TEMRA Th abs.	6.17 (3.11; 13.58)	18.46 (4.25; 36.31)	11.23 (6.24; 26.89)	1–3 *p* = 0.01
N Th%	29.88 (19.81; 36.00)	17.56 (15.72; 27.73)	33.39 (25.76; 39.09)	2-3 *p* = 0.03
N Th abs.	222.99 (145.88; 369.81)	143.50 (71.09; 317.64)	216.69 (140.38; 382.83)	n/s
CD4/CD8	2.01 (1.34; 2.44)	1.52 (1.16; 2.41)	1.96 (1.39; 2.17)	n/s

n/s: not significant.

**Table 6:** Impact of hypertonic disease on T cells populations in biological prosthesis recipients.

T cells populations	Healthy donors, *n* = 48	Hypertonic disease, *n* = 7	No hypertonic disease, *n* = 18	*p* value
CD3^+^%	78.27 (73.07; 82.52)	71.47 (62.71; 84.21)	69.26 (65.58; 74.10)	1–3 *p* < 0.001
CD3^+^ abs.	1419.31 (1126.25; 1735.91)	1515.78 (1003.36; 1643.88)	1204.92 (1004.40; 1327.53)	1–3 *p* = 0.04
CD3^+^CD8^+^%	25.80 (20.64; 33.20)	24.95 (23.07; 38.91)	22.28 (18.07; 27.32)	n/s
CD3^+^CD8^+^ abs.	462.37 (349.70; 647.87)	474.05 (360.40; 715.17)	374.22 (259.48; 485.60)	n/s
CM Tcyt%.	11.50 (8.70; 16.38)	6.41 (3.92; 7.50)	5.99 (3.93; 7.94)	1–3 *p* < 0.001 1-2 *p* = 0.006
CM Tcyt abs.	51.69 (36.70; 74.30)	30.38 (18.98; 46.34)	21.11 (13.17; 29.11)	1–3 *p* < 0.001 1-2 *p* = 0.01
EM Tcyt%	31.82 (18.80; 43.55)	33.21 (26.71; 42.60)	27.09 (15.97; 35.61)	n/s
EM Tcyt abs.	122.56 (74.29; 196.04)	162.12 (134.98; 201.94)	115.56 (55.09; 130.77)	2-3 *p* = 0.003
TEMRA Tcyt%	26.67 (19.58; 34.93)	49.41 (43.46; 58.83)	49.07 (40.68; 60.38)	1–3 *p* < 0.001 1-2 *p* < 0.001
TEMRA Tcyt abs.	104.32 (72.94; 188.42)	206.02 (140.19; 443.37)	173.57 (112.81; 284.02)	1–3 *p* < 0.001 1-2 *p* = 0.004
N Tcyt%	24.46 (16.51; 39.58)	7.53 (3.45; 12.06)	14.00 (10.98; 15.85)	1–3 *p* = 0.004 1-2 *p* < 0.001 2-3 *p* = 0.007
N Tcyt abs.	98.47 (56.29; 199.74)	33.37 (13.72; 43.46)	46.67 (39.46; 64.74)	1–3 *p* < 0.001 1-2 *p* < 0.001
CD3^+^CD4^+^%	48.06 (41.04; 52.58)	40.74 (32.23; 54.46)	41.81 (36.60; 48.37)	n/s
CD3^+^CD4^+^ abs.	835.92 (624.17; 1096.10)	817.20 (529.20; 1089.20)	705.31 (580.50; 948.64)	n/s
CM Th%	44.67 (38.42; 49.32)	46.91 (36.38; 48.95)	38.68 (35.23; 44.05)	1–3 *p* = 0.03
CM Th abs.	338.29 (269.64; 484.11)	297.29 (221.39; 468.69)	251.99 (227.73; 340.65)	1–3 *p* = 0.01
EM Th%	24.02 (17.54; 30.71)	33.21 (21.28; 36.49)	24.28 (17.82; 28.74)	n/s
EM Th abs.	197.64 (150.63; 229.90)	237.85 (155.53; 284.65)	162.54 (121.90; 187.07)	1–3 *p* = 0.04 2-3 *p* = 0.04
TEMRA Th%	0.82 (0.37; 1.67)	2.26 (0.94; 3.17)	1.62 (0.90; 2.49)	1–3 *p* = 0.009
TEMRA Th abs.	6.17 (3.11; 13.58)	18.46 (4.25; 33.43)	11.23 (6.24; 26.89)	1–3 *p* = 0.02
N Th%	29.88 (19.81; 36.00)	22.24 (16.52; 27.73)	35.46 (27.88; 39.09)	2-3 *p* = 0.01
N Th abs.	222.99 (145.88; 369.81)	159.70 (87.42; 317.64)	226.13 (140.38; 382.83)	n/s
CD4/CD8	2.01 (1.34; 2.44)	1.52 (1.16; 2.41)	2.01 (1.47; 2.17)	n/s

n/s: not significant.

**Table 7:** Impact of acute cerebrovascular accident to T cells populations in biological prosthesis recipients.

T cells populations	Healthy donors, *n* = 48	Acute cerebrovascular accident, *n* = 3	No acute cerebrovascular accident, *n* = 22	*p* value
CD3^+^%	78.27 (73.07; 82.52)	65.68 (62.34; 68.66)	71.84 (65.68; 76.38)	1-2 *p* = 0.005 1–3 *p* = 0.003
CD3^+^ abs.	1419.31 (1126.25; 1735.91)	853.84 (748.08; 1441.86)	1281.15 (1071.68; 1515.78)	n/s
CD3^+^CD8^+^%	25.80 (20.64; 33.20)	19.96 (18.93; 33.45)	24.52 (18.07; 27.32)	n/s
CD3^+^CD8^+^ abs.	462.37 (349.70; 647.87)	259.48 (227.16; 702.45)	413.12 (310.95; 597.77)	n/s
CM Tcyt%.	11.50 (8.70; 16.38)	3.05 (1.61; 3.93)	6.30 (4.21; 7.94)	1-2 *p* < 0.001 1–3 *p* < 0.001 2-3 *p* = 0.01
CM Tcyt abs.	51.69 (36.70; 74.30)	8.92 (4.17; 21.42)	26.35 (19.22; 31.72)	1-2 *p* < 0.001 1–3 *p* < 0.001
EM Tcyt%	31.82 (18.80; 43.55)	15.97 (14.01; 57.57)	32.65 (22.67; 38.57)	n/s
EM Tcyt abs.	122.56 (74.29; 196.04)	98.41 (41.43; 130.76)	120.99 (74.36; 162.12)	n/s
TEMRA Tcyt%	26.67 (19.58; 34.93)	71.45 (26.89; 76.42)	49.03 (43.46; 56.58)	1-2 *p* = 0.04 1–3 *p* < 0.001
TEMRA Tcyt abs.	104.32 (72.94; 188.42)	185.39 (61.08; 536.81)	198.48 (129.60; 332.12)	1–3 *p* = 0.002
N Tcyt%	24.46 (16.51; 39.58)	10.98 (6.52; 11.61)	13.49 (8.10; 15.83)	1-2 *p* = 0.03 1–3 *p* < 0.001
N Tcyt abs.	98.47 (56.29; 199.74)	28.49 (26.37; 45.79)	43.90 (33.37; 64.74)	1-2 *p* = 0.005 1–3 *p* < 0.001
CD3^+^CD4^+^%	48.06 (41.04; 52.58)	36.60 (34.28; 40.70)	43.02 (37.69; 53.87)	1-2 *p* = 0.03
CD3^+^CD4^+^ abs.	835.92 (624.17; 1096.10)	529.10 (439.20; 719.88)	723.73 (631.84; 979.38)	1-2 *p* = 0.03
CM Th%	44.67 (38.42; 49.32)	46.42 (34.21; 48.77)	39.46 (35.30; 45.49)	n/s
CM Th abs.	338.29 (269.64; 484.11)	245.60 (214.19; 246.27)	287.48 (227.73; 445.52)	1-2 *p* = 0.01 1–3 *p* = 0.04
EM Th%	24.02 (17.54; 30.71)	26.64 (22.92; 32.53)	25.82 (17.82; 29.66)	n/s
EM Th abs.	197.64 (150.63; 229.90)	140.95 (100.66; 234.17)	169.64 (135.15; 237.85)	n/s
TEMRA Th%	0.82 (0.37; 1.67)	1.18 (0.44; 14.03)	1.64 (0.94; 2.49)	1–3 *p* = 0.003
TEMRA Th abs.	6.17 (3.11; 13.58)	6.24 (1.93; 100.99)	11.71 (6.62; 26.89)	1–3 *p* = 0.008
N Th%	29.88 (19.81; 36.00)	25.76 (19.23; 27.88)	33.39 (24.50; 39.09)	n/s
N Th abs.	222.99 (145.88; 369.81)	136.29 (122.44; 138.43)	231.64 (159.70; 382.83)	2-3 *p* = 0.04
CD4/CD8	2.01 (1.34; 2.44)	1.93 (1.02; 2.03)	1.81 (1.39; 2.41)	n/s

n/s: not significant.

**Table 8:** Impact of GI-tract diseases on T cells populations in biological prosthesis recipients.

T cells populations	Healthy donors, *n* = 48	GI-tract diseases, *n* = 13	No GI-tract diseases, *n* = 12	*p* value
CD3^+^%	78.27 (73.07; 82.52)	66.98 (62.34; 76.34)	70.67 (66.32; 76.92)	1-2 *p* = 0.002 1–3 *p* = 0.02
CD3^+^ abs.	1419.31 (1126.25; 1735.91)	1230.80 (941.04; 1526.80)	1281.15 (1122.01; 1461.93)	n/s
CD3^+^CD8^+^%	25.80 (20.64; 33.20)	23.07 (18.02; 25.75)	26.84 (20.34; 36.02)	1-2 *p* = 0.04
CD3^+^CD8^+^ abs.	462.37 (349.70; 647.87)	360.40 (280.98; 464.44)	478.00 (327.14; 701.41)	1-2 *p* = 0.04
CM Tcyt%.	11.50 (8.70; 16.38)	6.44 (4.20; 7.94)	5.59 (3.21; 6.30)	1-2 *p* = 0.002 1–3 *p* < 0.001
CM Tcyt abs.	51.69 (36.70; 74.30)	22.28 (13.17; 38.49)	23.45 (19.23; 29.84)	1-2 *p* < 0.001 1–3 *p* < 0.001
EM Tcyt%	31.82 (18.80; 43.55)	32.94 (28.76; 42.60)	21.75 (13.0; 33.86)	*p* = 0.02
EM Tcyt abs.	122.56 (74.29; 196.04)	134.98 (116.62; 158.90)	108.44 (60.61; 147.64)	n/s
TEMRA Tcyt%	26.67 (19.58; 34.93)	46.69 (43.46; 49.49)	59.60 (42.14; 68.43)	1-2 *p* < 0.001 1–3 *p* < 0.001
TEMRA Tcyt abs.	104.32 (72.94; 188.42)	140.19 (129.60; 206.02)	277.78 (144.12; 461.98)	1–3 *p* = 0.002
N Tcyt%	24.46 (16.51; 39.58)	12.06 (8.10; 14.28)	13.43 (7.45; 25.74)	1-2 *p* < 0.001 1–3 *p* = 0.02
N Tcyt abs.	98.47 (56.29; 199.74)	40.57 (26.37; 54.33)	45.06 (33.97; 106.42)	1-2 *p* < 0.001 1–3 *p* = 0.01
CD3^+^CD4^+^%	48.06 (41.04; 52.58)	40.74 (36.19; 54.41)	41.91 (38.31; 45.28)	1–3 *p* = 0.02
CD3^+^CD4^+^ abs.	835.92 (624.17; 1096.10)	729.64 (579.04; 999.13)	705.31 (646.06; 882.92)	n/s
CM Th%	44.67 (38.42; 49.32)	44.05 (39.33; 47.82)	36.15 (34.83; 39.84)	1–3 *p* = 0.02
CM Th abs.	338.29 (269.64; 484.11)	330.65 (220.84; 449.51)	257.76 (245.93; 302.12)	1–3 *p* = 0.01
EM Th%	24.02 (17.54; 30.71)	25.64 (18.83; 28.86)	26.32 (15.97; 32.87)	n/s
EM Th abs.	197.64 (150.63; 229.90)	167.11 (144.86; 233.30)	166.95 (122.94; 254.08)	n/s
TEMRA Th%	0.82 (0.37; 1.67)	1.49 (0.94; 2.05)	2.31 (0.97; 4.01)	1–3 *p* = 0.005
TEMRA Th abs.	6.17 (3.11; 13.58)	10.34 (6.19; 15.86)	17.45 (6.58; 33.99)	1–3 *p* = 0.01
N Th%	29.88 (19.81; 36.00)	30.67 (24.50; 36.61)	33.39 (22.49; 41.32)	n/s
N Th abs.	222.99 (145.88; 369.81)	211.18 (140.38; 382.83)	219.48 (140.96; 326.85)	n/s
CD4/CD8	2.01 (1.34; 2.44)	2.06 (1.57; 2.98)	1.55 (1.09; 2.06)	n/s

n/s: not significant.
